# Effects of the timing of tourniquet release in cemented total knee arthroplasty: a systematic review and meta-analysis of randomized controlled trials

**DOI:** 10.1186/s13018-014-0125-0

**Published:** 2014-12-03

**Authors:** Wei Zhang, An Liu, Dongcai Hu, Yang Tan, Mohammed Al-Aidaros, Zhijun Pan

**Affiliations:** Department of Orthopedics, Second Affiliated Hospital, School of Medicine, Zhejiang University, Hangzhou, China; Department of Orthopedics, Zhongnan Hospital of Wuhan University, Wuhan, China

**Keywords:** Tourniquet, Knee, Arthroplasty, Blood loss, Complication

## Abstract

**Background:**

The aim of this study is to evaluate the effects of tourniquet release before wound closure for hemostasis or after wound closure in cemented total knee arthroplasty (TKA).

**Methods:**

We conducted a meta-analysis and review work on relevant clinical outcomes to evaluate the effects of the timing of tourniquet release in cemented TKA. Electronic databases were searched for relevant randomized controlled trials (RCTs) that compared outcomes of tourniquet release before wound closure for hemostasis with tourniquet release after wound closure. The methodological quality of each included RCT was assessed in terms of the 12-item scale. The meta-analysis was performed with STATA 12.0 software.

**Results:**

Eleven RCTs involving 651 patients with 670 TKAs were included in this meta-analysis. Of these, 332 patients (342 knees) were in an early tourniquet release group and 319 patients (328 knees) in the late tourniquet release group. The results showed that there were no significant differences in overt blood loss, hemoglobin drop, and blood transfusions, whereas the tourniquet release after wound closure might increase the risks of overall complications and major complications.

**Conclusions:**

Tourniquet release before wound closure for hemostasis might reduce the rate of complications, but it could not limit overall blood loss. The current evidences are not enough to indicate that tourniquet release before wound closure is superior to its release after wound closure in cemented TKA.

## Background

Total knee arthroplasty (TKA) is performed with a tourniquet. It is widely accepted that the use of a tourniquet in TKA contributes to reduce intraoperative blood loss, to allow better visualization, and to ease cementing of the prosthesis.

However, there are controversies on the optimal timing of tourniquet application, which might exert important influence on clinical outcomes [1]. The most common tourniquet application strategies in TKA are tourniquet release before wound closure for hemostasis and tourniquet release after wound closure. Both strategies have their pros and cons. Some researchers believed that tourniquet release before wound closure for hemostasis might be a valid and reasonable option, for patients had less perioperative pain [2], better functional recovery [2,3], less blood loss [3], and lower risk of complications [4], especially regarding to the presence of reoperations due to serious vascular injury [5]. Meanwhile, some authors claimed that if the tourniquet release after wound closure, it could alter patellofemoral tracking, which could result in unnecessary lateral retinacular release and even patellar instability [6,7]. In contrast, some authors recognized that it was unnecessary to release the tourniquet before wound closure for hemostasis. They found that similar blood loss [8-12], risk of complications [13-15], and functional recovery [8,13] during TKA with or without release of the tourniquet before wound closure for hemostasis. Furthermore, the duration of hemostasis would increase surgical time and anesthetic time [5,15,16], which might increase unnecessary risks and medical costs.

Based on the recent survey, United Kingdom, Australia, Sweden, and New Zealand registry data showed greater usage of cemented than non-cemented fixation in TKA due to lower failure rates [17]. Moreover, the main aim of using a tourniquet is achieving superior cementation. However, several studies confirmed that there were some different clinical outcomes between cemented and non-cemented TKA, including blood loss and rate of complications, etc. [18-21]. To clarify this and decrease heterogeneity, several randomized controlled trials (RCTs) concerning the optimal timing of tourniquet application have been published [2,3,5,8,9,14-16,22-24], but consensus, as yet, was not attained. To the best of our knowledge, all the previous meta-analysis included cemented and non-cemented prosthesis. As a result, in order to provide an evidence for clinical practice, it is necessary to have a latest meta-analysis to evaluate and summarize this issue, especially in cemented TKA. The purpose of our study is to evaluate the effects of tourniquet release before and after wound closure in cemented TKA.

## Methods

This meta-analysis was preformed according to the Preferred Reporting Items for Systematic Reviews and Meta-Analyses guidelines (the PRISMA statement) [25].

### Retrieve strategies

Electronic databases, including Medline, Embase, and Cochrane Central Register of Controlled Trails and ISI Web of Knowledge were searched by two independent researchers (W.Z. and A.L), which were published up to September 15, 2014. The following search terms were used: ((total knee arthroplasty) OR total knee replacement) AND tourniquet. Meanwhile, reference lists of the relevant articles were also retrieved for any additional relevant studies. Languages were not restricted in the search.

### Inclusion criteria and exclusive criteria

We identified studies according to the following inclusion criteria:

1. Target population: individuals underwent primary TKA
2. Intervention: a comparison between tourniquet release before wound closure for hemostasis (early release group) and tourniquet release after wound closure (late release group)
3. Outcome: trials that reported blood loss, complications, or functional rehabilitation (at least one desirable outcome)
4. Methodological criterion: a prospective RCT
5. Cemented prosthesis

The following criteria were used for exclusion:

1. Revision TKA and complicated TKA
2. Animal studies and cadaver studies
3. Non-cemented and hybrid prosthesis for implanting
4. Unicompartmental knee arthroplasty
5. Tourniquet inflated only during cementation of the implants
6. Tourniquet release before prosthesis was inserted; hemostasis was attained and then reinflated

### Data extraction

Two authors (W.Z. and A.L.) extracted relevant data independently, including demographic characteristic (sample size, average age, body mass index (BMI)), study design, tourniquet pressure, drainage, anticoagulant, overt blood loss, overall blood loss, hemoglobin drop, volume of transfusion, rate of transfusion, and complications (the number of reoperations, the number of thrombotic events, etc.). Overt blood loss was defined as intraoperative blood loss and volume of wound drainage. The overall blood loss was calculated by conventional formula [3] (Gross formula, modified Gross formula, etc.) based on hemoglobin or hematocrit. Reoperations included vessel injuries, infections, wound dehiscence and hematomas that required drainage and/or debridement, and serious flexion contraction that required manipulation with the patient under anesthesia. We defined major complications as reoperations and thrombotic events. In addition, we also tried to contact the authors of the eligible studies to ask for relevant original data for this meta-analysis.

### Quality assessment

The methodological quality of each included RCT was assessed by two independent researchers (W.W. and A.L.) in terms of the 12-item scale [26]. Each item was scored “Yes” or “No” with a maximum score of 12 “Yes”. Any trial with a score of 7 “Yes” or more was considered high quality, more than 4 but no more than 7 was considered moderate quality, and no more than 4 was considered low quality. If disagreements were encountered, they were evaluated by the means of a kappa test and were resolved by discussion with the corresponding author (Z.P.).

### Statistical analysis

The meta-analysis was conducted with STATA 12.0 (Stata Corp, College Station, Texas). For continuous outcomes, a weighted mean difference (WMD) and 95% confidence interval (CI) was used. For dichotomous data, a risk ratio (RR) and 95% CI were calculated as the summary statistics. The statistical heterogeneity was tested with the chi-square test and *I*^2^*. I*^2^ < 25% was considered low statistical heterogeneity; *I*^2^ < 50%, moderate statistical heterogeneity; and *I*^2^ < 75%, high statistical heterogeneity [27]. If *P* > 0.1 and *I*^2^ < 50%, the fixed effects model was used; otherwise, we used the random effects model. Egger’s test and Begg’s test was performed to assess publication bias. The Grading of Recommendations Assessment, Development and Evaluation (GRADE) approach was used to each pooling of outcomes performed to determine the quality of evidence [28].

## Results

### Study selection

**Figure 1:**
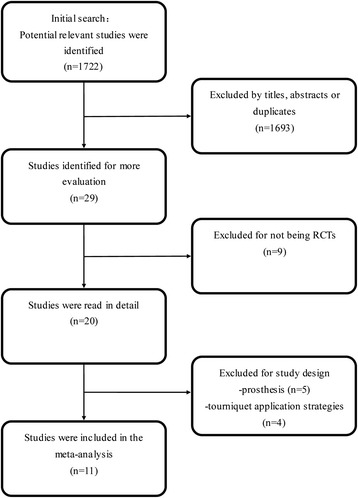
**Flow chart summarizing the selection process of randomized control trials (RCTs).**

A flowchart of studies selection was in Figure 1. According to our search strategy, 1,722 potential relevant articles were identified initially: 475 from Medline, 448 from Embase, 157 from the Cochrane database, and 642 from ISI Web of Knowledge. Of these, 1,693 studies were excluded for the titles, abstracts, or duplicates. Then, 29 studies were retrieved in full text, including 27 English studies and 2 German studies. These two German studies were translated by a professional medical translator [9,29]. Among these 29 studies, 18 studies were excluded, of which 9 studies were not RCTs [11-13,29-33]; 5 studies involved non-cement TKA, hybrid TKA, or unicompartmental knee arthroplasty [20,34-37]; 2 studies included the use of tourniquet only during cementation of the implants [38,39]. One study included simultaneous bilateral TKA, in which one knee was operated with tourniquet release early and the other knee with tourniquet late for all of the patients included in the study [40]. In addition, tourniquet was released before prosthesis was inserted; hemostasis was attained and then inflated again in another study [41]. Therefore, it was also excluded. Finally, 11 RCTs were included in this meta-analysis [2,3,5,8,9,14-16,22-24]. There was an excellent interrater agreement between investigators on eligibility (*Κ* = 1.0).

### Study characteristics

The characteristics of 11 included studies were in Table 1. Eleven studies included 1 German study and 10 English studies. The dataset involved 651 patients with 670 knees, of which 332 patients (342 knees) had tourniquet release before wound closure for hemostasis and 319 patients (328 knees) had tourniquet release after wound closure. Five patients (two in early release group, three in late release group) were lost to follow-up [3]. Baseline demographics (the average age, BMI, gender ratio) between the two groups were comparable. The tourniquet cuff pressure ranged from 220–400 mmHg.

**Table 1:** **Study characteristics**

**Study**	**Publication year**	**Group size**		**Total size**	**Lost to follow-up**		**Mean age (years)**		**BMI (kg/m** ^**2**^ **)**		**Drainage**	**Cuff pressure**	**Anticoagulation**	**The duration of tourniquet (minutes)**	
		**Early**	**Late**		**Early**	**Late**	**Early**	**Late**	**Early**	**Late**				**Early**	**Late**
Kvederas et al. [3]	2013	14	15	36	2	3	68.2	67.3	32.0	31.1	Y	N.A.	Heparin	37	60
Leão et al. [24]	2013	20	20	40	0	0	65.3	65.4	N.A.	N.A.	Y	350	N.A.	N.A.	N.A.
Dutton et al. [23]	2012	28	20	48	0	0	N.A.	N.A.	N.A.	N.A.	Y/N	N.A.	Enoxaparin	N.A.	N.A.
Yavarikia et al. [5]	2010	33	22	55	0	0	64	68	N.A.	NA	Y	220–275	Heparin	63	78
Steffin et al. [14]	2009	17	20	37	0	0	66.9	65.6	33.4	32.5	Y	N.A.	Enoxaparin	N.A.	N.A.
Hernandez et al. [16]	2008	21	22	43	0	0	75.9	76	N.A.	N.A.	Y	N.A.	N.A.	N.A.	N.A.
Hersekli et al. [15]	2004	36	40	76	0	0	65	68	N.A.	N.A.	Y	350–400	Coumadin	79	83
Schuh et al. [9]	2003	35	35	70	0	0	N.A.	N.A.	N.A.	N.A.	Y	N.A.	Enoxaparin	72	102
Widman et al. [8]	1999	46	39	81	0	0	N.A.	N.A.	N.A.	NA	Y	300–350	Heparin	N.A.	N.A.
Barwell et al. [2]	1997	44	44	88	0	0	N.A.	N.A.	N.A.	NA	Y	Twice^a^	N.A.	N.A.	N.A.
Newman et al. [22]	1979	38	42	80	0	0	N.A.	N.A.	N.A.	NA	N.A.	N.A.	Heparin	N.A.	N.A.

*N.A.* not available.

^a^Twice the systolic blood pressure.

### Study quality

According to 12-item scale, the methodological quality of each included RCTs was evaluated (Table 2). The value of weighted kappa for the agreement on these studies between reviewers was excellent (*Κ* = 0.80). All the studies were high quality except for one study [23]. The average score for the quality of studies was 7.82 points (of 12 points), and the standard deviation was 1.25 points. Six studies used randomized method adequately. Randomization allocation was concealed appropriately in three studies. Blinding method was applied to three studies.

**Table 2:** **Study quality**

**Study**	**Randomized adequately** ^**a**^	**Allocation concealed**	**Patient blinded**	**Care provider blinded**	**Outcome assessor blinded**	**Acceptable drop-out rate** ^**b**^	**ITT analysis** ^**c**^	**Avoided selective reporting**	**Similar baseline**	**Similar or avoided cofactor**	**Patient compliance**	**Similar timing**	**Quality** ^**d**^
Kvederas et al. [3]	Yes	Yes	Yes	No	Yes	Yes	No	Yes	Yes	Yes	Yes	Yes	High
Leão et al. [24]	No	No	No	No	No	Yes	Yes	Yes	Yes	Yes	Yes	Yes	High
Dutton et al. [23]	No	No	Yes	No	No	Yes	Yes	Unclear	Unclear	Yes	Yes	Unclear	Moderate
Yavarikia et al. [5]	No	Yes	Unclear	No	Unclear	Yes	Yes	Yes	Yes	Yes	Yes	Yes	High
Steffin et al. [14]	Yes	No	No	No	Unclear	Yes	Yes	Yes	Yes	Yes	Yes	Yes	High
Hernandez et al. [16]	Yes	No	No	No	Unclear	Yes	Yes	Yes	Yes	Yes	Yes	Yes	High
Hersekli et al. [15]	No	Yes	Unclear	No	Unclear	Yes	Yes	Yes	Yes	Yes	Yes	Yes	High
Schuh et al. [9]	Yes	No	Unclear	No	Unclear	Yes	Yes	Yes	Yes	Yes	Yes	Yes	High
Widman et al. [8]	Yes	No	No	No	Unclear	Yes	Yes	Yes	Yes	Yes	Yes	Yes	High
Barwell et al. [2]	Yes	No	Yes	No	No	Yes	Yes	Yes	Yes	Yes	Yes	Yes	High
Newman et al. [22]	No	No	Unclear	No	Unclear	Yes	Yes	Yes	Yes	Yes	Yes	Yes	High

^a^Only if the method of sequence made was explicitly introduced could get a ‘Yes’.

^b^Drop-out rate <20% could get a ‘Yes’, otherwise ‘No’.

^c^ITT = intention-to-treat, only if all randomized participants were analyzed in the group they were allocated to could receive a ‘Yes’.

^d^“Yes” items more than 7 means ‘High’; more than 4 but no more than 7 means ‘Moderate’; no more than 4 means ‘Low’.

### Meta-analysis results

#### Hemoglobin drop

**Figure 2:**
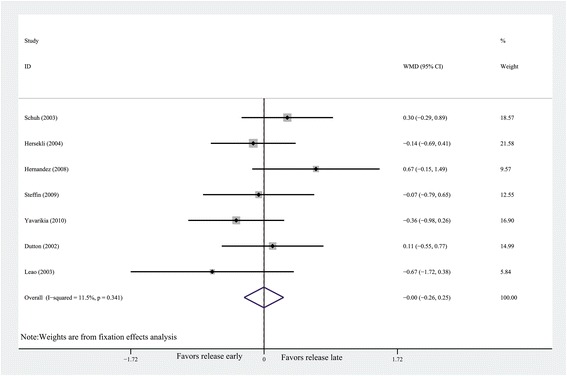
**Forest plot for hemoglobin drop.** CI, confidence interval; WMD, weighted mean difference.

The forest plot shows that there was no difference between early tourniquet release group and late tourniquet release group in TKA (*n* = 369, WMD = 0; 95% CI −0.26 to 0.25, *P* = 0.98; heterogeneity *P* = 0.341, *I*^2^ = 11.5%) (Figure 2).

#### Rate of transfusion

**Figure 3:**
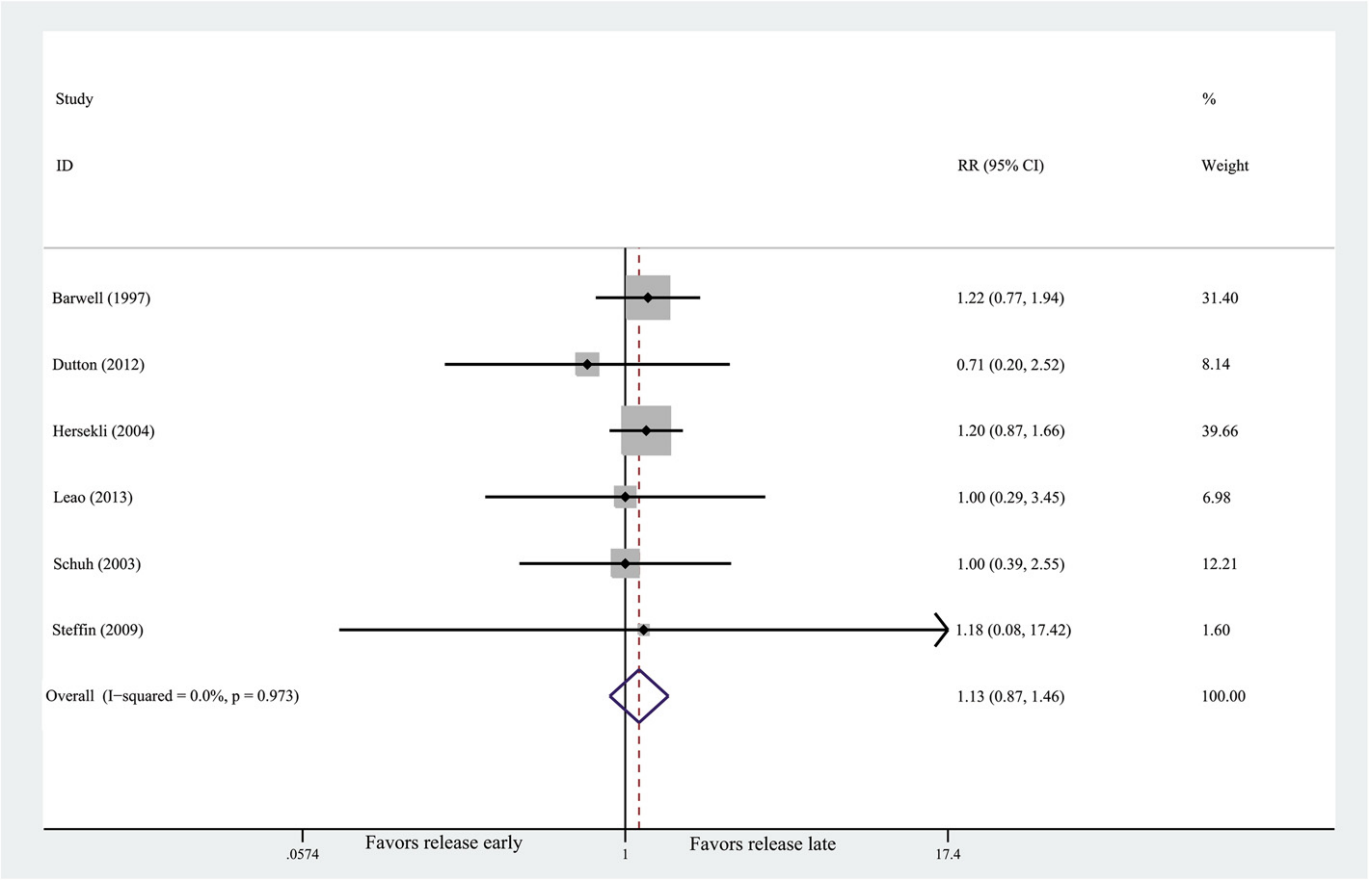
**Forest plot for the rate of transfusion.** CI, confidence interval; RR, risk ratio.

The pooling of outcomes demonstrates that no difference was detected in the rate of transfusion (*n* = 369, RR = 1.13, 95% CI 0.87 to 1.47, *P* = 0.35; heterogeneity *P* = 0.973, *I*^2^ = 0%) (Figure 3).

#### Volume of transfusion

No difference were also observed in volume of transfusion between the two groups (*n* = 259, WMD = 5.27, 95% CI −30.75 to 41.29, *P* = 0.77; heterogeneity *P* = 0.62, *I*^*2*^ = 0%).

#### Overt blood loss

The forest plot shows that no difference was observed in overt blood loss when tourniquet was released before wound closure or after wound closure (*n* = 183, WMD = 37.53, 95% CI −24.72 to 99.78, *P* = 0.24; heterogeneity *P* = 0.95, *I*^*2*^ = 0%).

#### Overall complications

**Figure 4:**
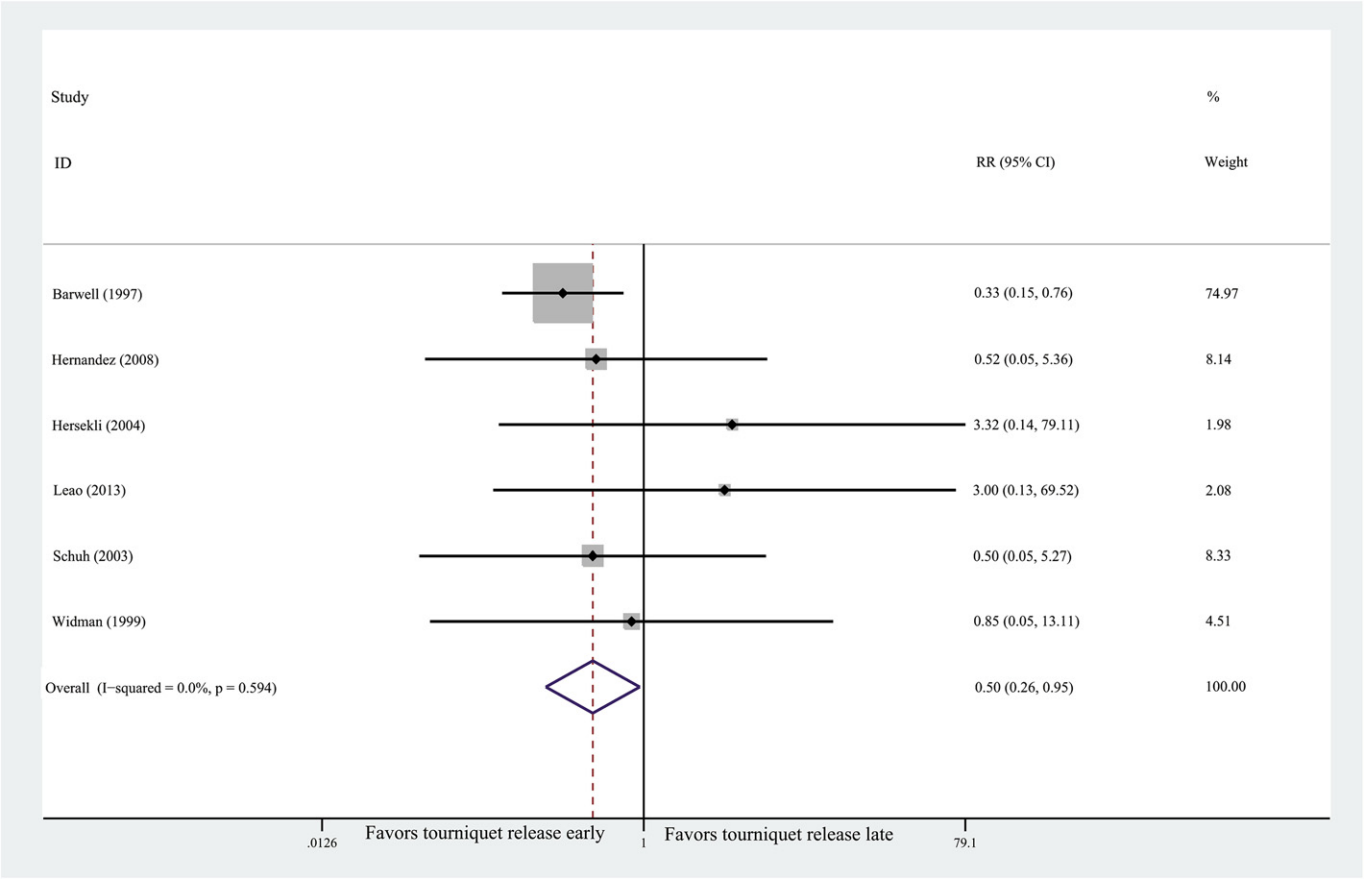
**Forest plot for overall complications.** CI, confidence interval; RR, risk ratio.

Full details of complications were summarized in Table 3. The forest plot shows that the rate of overall complications were significantly lower in the early release group than those in the late release group (*n* = 468, RR = 0.50, 95% CI 0.26 to 0.95, *P* = 0.034; heterogeneity *P* = 0.594, *I*^*2*^ = 3%) (Figure 4).

**Table 3:** **Complications results**

**Complication**	**No. of studies**	**No. of patients**		**No. of complications (%)**	
		**Release early**	**Release late**	**Release early**	**Release late**
Minor wound complications^a^	4	161	158	9 (5.59)	13 (8.23)
Flexion contracture	2	65	66	1 (1.54)	3 (4.55)
Thrombotic events	2	81	74	0 (0)	3 (4.05)
Reoperation^b^	3	85	86	2 (2.35)	7 (8.14)
Major complications^c^	5	166	160	2 (1.20)	10 (6.25)
Total	8	233	235	11 (8.58)	23 (9.79)

^a^Minor wound complications included wound oozing, erythema, cellulitis, minor dehiscence, superficial infection, etc.

^b^Vessels injury, infection, wound dehiscence, and hematomas that required drainage and/or debridement and knee stiffness that required manipulation with the patient under anesthesia.

^c^Reoperations and thrombotic events.

#### Major complications

**Figure 5:**
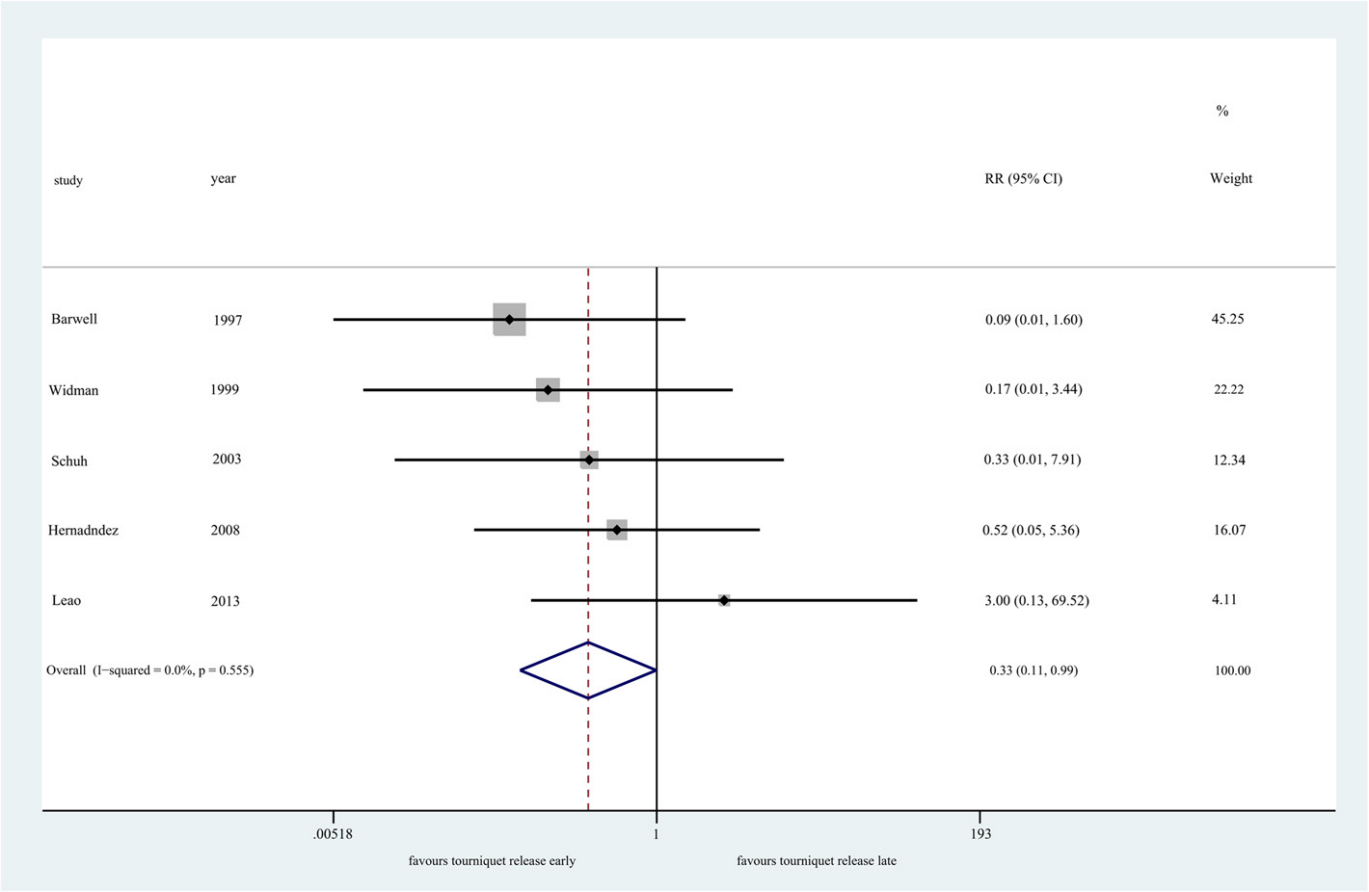
**Forest plot for major complications.** CI, confidence interval; RR, risk ratio.

Major complications included the reoperations and thrombotic events. They were also in Table 3. Tourniquet release before wound closure could reduce the risk of major complications (*n* = 326, RR = 0.33, 95% CI 0.11 to 0.99, *P* = 0.049; heterogeneity *P* = 0.555, *I*^*2*^ = 0%) (Figure 5). Greater incidence of reoperations and thrombotic events were detected in the early release group compared with those in the late release group (1.20% vs. 6.25%).

### GRADE analysis

According to the GRADE approach, the quality of the evidence was moderate for maximum hemoglobin drop and the rate of transfusion, low for the rate of overall complications, very low for the volume of transfusion, the rate of major complications.

## Discussion

The most important finding of the meta-analysis was that there were no significant differences in the hemoglobin drop, overt blood loss, rate of transfusion, and volume of transfusion between tourniquet release before wound closure for hemostasis and tourniquet release after wound closure in TKA. The risks of overall complications and major complications could be decreased due to tourniquet release before wound closure for hemostasis.

For blood loss, the result demonstrates that there was no difference in hemoglobin drop between the two groups. Those results were accordance with other studies [10-12,20,30]. The hemoglobin drop, rather than overt blood loss, is one of the most objective clinical outcomes to reflect the overall blood loss. Furthermore, similar rate of transfusion and volume of transfusion were detected between the two groups. These phenomena indicated similar overall blood loss between the two groups. Likewise, a RCT also shows that no significant difference was observed in calculated blood loss (proposed by Gross) between the two groups in cemented TKA [9].

In fact, rapid reactive hyperemia and increased fibrinolytic activity occurs after tourniquet release, leading to ongoing bleeding from cut cancellous bone and contributing to major perioperative blood loss [32,41]. It has been demonstrated that a local compressive effect is the primary method to control the bleeding [12,32]. Although there was less intraoperative blood loss when tourniquet was released after wound closure, the benefit might be counteracted by more postoperative blood loss, for the surgeons were unable to identify and cauterize small bleeding vessels during the operation [30]. Meanwhile, tourniquet release after wound closure might result in the unnecessary lateral retinacular release, and lateral retinacular release was an independent risk factor associated with the rate of transfusion following TKA [42].

As for complications, this study shows that tourniquet release before wound closure for hemostasis reduced the risks of overall complications and major complications. Likewise, consistent results were found in several studies [12,30,35,36]. The possible reasons for those phenomena could be as follows. On the one hand, the prolonged duration of tourniquet used might be a crucial factor for complications, which suggests longer ischemic time for tissues. Tourniquet release after wound closure could cause more excessive inflammation and muscle damage [30]. Moreover, several studies have demonstrated an increased rate of complications, including wound oozing, nerve injury, when longer tourniquet times have been used [43-45]. Every additional 10 min of tourniquet time was associated with an increased risk for complications [46]. Nerve injuries occurred with an odds ratio of 2.8 for each 30 min of tourniquet time [47]. Sherman et al. also reported that the use of a tourniquet for longer than 40 min placed the patient at moderate risk and that tourniquet use longer than 60 min placed the patient at high risk of developing a complication [48]. As a result, it is crucial to minimize the tourniquet time. On the other hand, tourniquet might alter the patellofemoral tracking when it was released after wound closure. It might influence the surgeons’ judgments, thereby leading to the unnecessary performance of a lateral release, which might have a detrimental effect on patellar viability and could increase the incidence of hematomas requiring drainage and wound edge avascularity [49-51]. Nevertheless, the examinations, such as no thumb test, for patellofemoral tracking could be properly achieved when tourniquet was released before wound closure, which would avoid those problems. Last but not the least, tourniquet release for hemostasis before wound closure would be a practical way to determine the major vascular damage. According to a recent epidemical survey, the rate of acute arterial complications was about 0.1% (37 of 39,196 TKAs) from 1989 to 2012 [52]. Although major vascular injury in TKA is very rare, early recognition and expeditious management of these injuries are critical for successful outcomes. Therefore, the major complications occurred rarely in TKA, but tourniquet release after wound closure increase the duration of tourniquet and could not detect the specific injury timely, thereby possibly leading to higher risk.

As regards to subjective performance and functional recovery, there is not enough data to be combined and analyzed. Of those, two RCTs reported that better subjective performance and earlier functional recovery were observed at early postoperative follow-ups in early release group [2,3]. Nevertheless, another RCT found no difference in the range of motion between the two groups at the first postoperative follow-up 2–3 months after TKA [8]. Then, some researchers begin to focus on whether the limited use of a tourniquet (using a tourniquet only during the cementation) in cemented TKA facilitate function recovery. As the result of Fan et al.’s trial, the limited use of a tourniquet provided the benefit of decreased limb swelling and knee joint pain while much more blood loss was detected [53]. Similarly, Huang et al. and others also found that releasing the tourniquet after wound closure increase more excessive inflammation and muscle damage [30,54]. For a more objective clinical outcome, more RCTs with high quality on those issues and longer follow-up period are required.

The current meta-analysis had several strengths. Based on a thorough search of literature, all 11 included studies were randomized controlled trials, 10 of which were high methodological quality. All outcomes of this meta-analysis existed low heterogeneity (*I*^2^ < 50%); thereby, the outcomes of this study would be much more reliability, which could be proved by sensitivity analysis. However, previous meta-analysis [4,55] included several non-RCTs, which might be a reason for different outcomes. In addition, as we know, the main aim for using a tourniquet is achieving superior cementation. Consequently, it is not very important to use a tourniquet in non-cemented TKA compared with cemented TKA. Unsolidified cement could limit bleeding from cancellous bone by a tamponade effect [56]; thus, less blood loss would be detected in cemented TKA. Ranawat et al.’s meta-analysis indicated greater functional outcomes, lower revision rates, and less patellofemoral complications among cemented TKAs [21]. Therefore, the interferences of non-cemented prosthesis for the final clinical outcomes were excluded in this study. To the best of our knowledge, this study was the first meta-analysis concerning cemented TKA.

There are also some limitations in this study. To begin with, publication bias might exist in this study using Begg’s test (*P* = 0.024) and Egger’s test (*P* = 0.005), for the sample size was small. Nevertheless, it was minimized by comprehensive search and rigorous assessments of methodology. Meanwhile, we included more RCTs concerning cemented TKAs than previous meta-analysis. Secondly, some confounding factors such as the timing of drain clamping, method of thromboembolic prophylaxis, the type of postoperative compressive dressing, the type of rehabilitation program and tourniquet pressure might influence the outcomes. Some of those managements were in Table 1. Moreover, the inflating pressure and duration of application of tourniquet were two crucial factors for complications. However, there was not enough data in the included studies to analyze. Thirdly, postoperative subjective performance and functional recovery were poorly assessed. Meanwhile, the follow-up period was too short. Hence, to evaluate the advantages and disadvantages of different tourniquet strategies in cemented TKA properly, further RCTs with well-designed are required.

## Conclusions

Based on currently available evidences, tourniquet release for hemostasis before wound closure could reduce the risk of overall complications and major complications compared with tourniquet release after wound closure in cemented TKA, although similar blood loss was observed. Considering the relatively small sample size, short follow-up period, and lack of assessment of postoperative subjective performance and functional recovery, the current evidences are not enough to indicate that tourniquet release before wound closure is superior to its release after wound closure in cemented TKA.
